# Deciphering the role of miR-187-3p/LRFN1 axis in modulating progression, aerobic glycolysis and immune microenvironment of clear cell renal cell carcinoma

**DOI:** 10.1007/s12672-022-00523-z

**Published:** 2022-07-07

**Authors:** Wenhao Xu, Wangrui Liu, Aihetaimujiang Anwaier, Xi Tian, Jiaqi Su, Guohai Shi, Shiyin Wei, Yuanyuan Qu, Hailiang Zhang, Dingwei Ye

**Affiliations:** 1 Department of Urology, Fudan University Shanghai Cancer Center, Shanghai Medical College, Fudan University, Dong’an Road 270, Shanghai, 200032 People’s Republic of China; 2 Department of Interventional Oncology, Renji Hospital, Shanghai Jiao Tong University School of Medicine, Shanghai, 200127 People’s Republic of China; 3 Affiliated Hospital of Youjiang Medical University for Nationalities, Baise, 533000 People’s Republic of China

**Keywords:** Clear cell renal cell carcinoma, microRNA, miR-187-3p, LRFN1, Biomarker, Tumor microenvironment (TME), Intra-tumoral heterogeneity

## Abstract

Clear cell renal cell carcinoma (ccRCC) is one of the most common malignant genitourinary cancers with high recurrence risk worldwide. Recently, multi-omics data facilitate obtaining a molecular landscape of tumor development, and were implemented to affect pathogenesis, phenotype, and prognosis of ccRCC. In this study, after screening for differential expressed microRNAs based on multiply datasets, we tested expression levels and prognostic value of miR-187-3p in ccRCC samples, and transfected miR-187-3p mimics or negative controls into ccRCC cells. Up-regulation of miR-187-3p restrains proliferation, migration and promotes apoptosis ability in human ccRCC A498 and 786O cells. In addition, Luciferase reporter assay revealed that miR-187-3p directly targets LRFN1-3’-UTR and negatively modulates LRFN1 expression. LRFN1 rescues proliferation and invasion capacities after miR-187-3p mimic transfection in vitro and in subcutaneous xenograft models. We further performed deep-sequencing technology and bioinformatics analyses to evaluate the biological functions and potential clinical implications of LRFN1 expression in ccRCC. Interestingly, LRFN1 could serve as an independent and potential biomarker for prognosis in over 1000 patients with ccRCC from multiply independent cohorts. Besides, the up-regulated LRFN1 expression prominently promoted intra-tumoral heterogeneity and immune-infiltrating microenvironment, represented by elevated M2 macrophage infiltration, CD8^+^ T cells activity and PD-L1 expression. In conclusion, this study revealed the tumor-specific and immunological role of miR-187-3p/LRFN1 axis in the progression and reshaping of tumor immune microenvironment of ccRCC.

**Supplementary Information:**

The online version contains supplementary material available at 10.1007/s12672-022-00523-z.

## Introduction

Renal cell carcinoma (RCC) is one of the most common genitourinary tumors in the world, accounting for 3% of all malignant tumors, and its incidence is increasing at a rate of 3% every year [1, 2]. Additionally, studies have shown an estimated 66,800 new cases and 23,400 deaths in 2015 in China, and the incidence rate in major domestic cities of China has reached more than 8/100,000, which is more than double that of 10 years ago [3, 4]. Clear cell renal cell carcinoma (ccRCC) is the most common (80–85%) and malignant pathological subtype of RCC in adults [5]. Despite extensive research into the mechanisms of carcinogenesis and progressive progression, the etiology of kidney cancer remains to be elucidated [5–7]. Currently, ccRCC is characterized by its heterogeneous and complex tumor microenvironment (TME), and it is difficult to identify high-risk patients for recurrence after therapeutic nephrectomy due to uncertain description of intra-tumoral heterogeneity and lack of effective biomarkers [8–10]. Considering high morbidity and mortality of ccRCC, it is critical to reveal the causes of aggressive malignancy and explore molecular biomarkers for early diagnosis, prognostic prediction and guiding personalized treatment strategies [11].

MicroRNAs (miRNAs) are a class of noncoding RNAs with a length of 18–22 nucleotides that do not have transcriptomic biological functions [12, 13]. There are approximately 2500 species of miRNAs involved in the regulation and control activities in adults [13]. The large number of reports have shown that miRNAs are playing an important role in the occurrence and development of tumors such as renal cell carcinoma [14], prostate cancer [15], breast cancer [16], liver cancer [17], colorectal cancer [18–21], and lung cancer [22]. Furthermore, many previous studies revealed that over- or under-expression of the human-specific miRNAs could regulate tumor cell progression, invasion and metastasis, drug resistance, angiogenesis or apoptosis by negatively regulating the expression of mRNAs [23, 24]. Recent studies have shown that miR-187 is significantly downregulated in many types of human malignancies [25–27]. For instance, miRNA-187-3p mitigates proliferation, migration, invasion, and promoting apoptosis of non-small cell lung cancer through targeting oncogenic BCL-6 [27].

RNA sequencing (RNA-Seq) has been widely utilized to detect genetic alterations across the genome and generate large amounts of data [28]. With the development of bioinformatics and multi-omics data, the bioinformatics algorithms could help cluster molecular phenotypes, distinguish molecular pathological features and identify effective biomarkers for tumors [29, 30]. In this study, we hypothesized that miR-187-3p may play a role in suppressing tumors, and mediates malignancy and tumor immune microenvironment (TIME) by targeting Leucine Rich Repeat and Fibronectin Type III Domain Containing 1 (LRFN1). For verification, in vitro cellular experiments, in vivo animal assays and in silico bioinformatics prediction algorithms were performed. These explorations might provide novel theoretical and therapeutic targets for treatment responses for ccRCC.

## Materials and methods

### Screening for transcriptional microRNAs expression profiles

NCBI- Gene Expression Omnibus (GEO) is regarded as a free public database of transcriptional expression profile and we obtained the differential expressed miRNAs (DEmiRNAs) profile from GSE12105, GSE55138 and GSE109368 investigating ccRCC and normal kidney tissues. The data of GSE12105 were obtained from the GPL6955 Agilent-016436 Human miRNA Microarray 1.0 (Feature Number version) and came from 12 kidney tumors and 12 normal kidney tissue samples [31]. Similarly, the data of GSE55138 were based on the GPL14613 ([miRNA-2] Affymetrix Multispecies miRNA-2 Array) [32]. The gene microarray data were collected from 16 clear cell renal carcinoma and 9 adjacent non-tumor samples. The GSE109368 data were obtained from the GPL18573 [Illumina NextSeq 500 (*Homo* *sapiens*)]. 12 primary ccRCC cancer tissues and 12 normal renal tissues were analyzed for the GSE109368 dataset.

### Standardization and elucidation of DEmiRNAs

After downloading matrix files for each GEO dataset, we utilized a Robust Multi-Array Average (RMA) algorithm in order to adjust data for background signals, and to conduct quantile normalization and final oligonucleotides-per-transcript summarization with a median polish algorithm. The R “Impute” package was used to impute missing values based on a k-nearest neighbor approach. Bayes methods were used to adjust for batch effects with the sva R package, as published previously, with R v3.6.1 being used for all analyses [33]. DEmiRNAs were identified via comparing ccRCC and normal kidney tissue gene expression profiles based on adjusted P-values, fold change values, and Benjamini and Hochberg FDR values. An online Venn diagram program was then used to identify shared DEmiRNAs among these three datasets (Supplementary Table 1).

### Cell culture

To elucidate expression difference of miR-187-3p between ccRCC and normal kidney samples, we included four genotyped ccRCC cell lines (A498, 786O, Caki1, Caki2) and the conventional normal human kidney cell line HK-2 cells from American Type Culture Collection (ATCC, Manassas, VA, USA). Cells were cultured in Dulbecco’s modified Eagle’s medium (DMEM; Gibco, Shanghai, China). All media were supplemented with 10% FBS (v/v), 2 mM L-glutamine and 100U/ml penicillin/streptomycin. Cells were cultured at 37 °C in a humidified atmosphere with 5% CO_2_.

### Total RNA extraction and quantitative real-time PCR analysis

For the detection of miR-187-3p expression, all miRNA extractions were performed using the miRNeasy Kit (Cat. No. 217084, Qiagen), all in strict accordance with the manufacturer’s instructions. A total of 20 paired tumor and normal kidney tissues were obtained from Fudan University Shanghai Cancer Center (FUSCC, Shanghai) to assess differential expression of miR-187-3p. MiR-187-3p sequences, forward, 5′-GCA GGA ACA TCT CCG GCT C-3′ and reverse, 5′-GCT AGG AGC TGT CCT TTA GGA-3′. cDNA was synthesized using the miScript II RT Kit (Cat. No. 331231, Qiagen). The expression of miRNA was analyzed using the miScript SYBR Green PCR Kit (Qiagen) with RNU6 (miRNA) as an endogenous control. Total RNA from harvested cell lines was isolated by Trizol (Invitrogen, Carlsbad, CA), and qRT-PCR was performed using SYBR^®^ Premix Ex TaqTM (TaKaRa) according to manufacturer’s protocol. The primers pairs were: LRFN1 forward, 5′‐TGG TCC GTC TGG ACA TGA C‐3′, LRFN1 reverse, 5′‐CAG GTC TCT AAG TCG TCC TCG‐3’; GAPDH forward, 5′‐CCA TGG AGA AGG CTG GGG‐3′, GAPDH reverse, 5′‐CAA AGT TGT CAT GGA TGA CC‐3′, The mRNA expression level was normalized to beta-Actin and replicated in triplicate according to the manufacturer’s instructions. The relative expression quantity was calculated using the 2^−ΔΔCt^ method.

### Western blotting analysis

Cells were collected and lysed using RIPA buffer (50 mM Tris, pH 7.4, 150 mM NaCl, 1%TritonX-100, 1% sodium deoxycholate, 0.1% SDS, 2 mM sodium pyrophosphate, 25 mM β-glycerophosphate, 1 mM EDTA, 1 mM Na3VO4, and 0.5 ug/ml leupeptin). Cell lysates were separated by SDS-PAGE, and electrophoretically transferred to NC membranes (Lot. G9597136, GE Health Care Life Science). After blocking using 5% nonfat milk in PBST, the membranes were incubated at 4 °C overnight with indicated antibodies. Primary antibodies were from commercial sources: anti-LRNF1 (Abcam, ab106365), and anti-Actin (Zen bioscience, #220529). The secondary antibodies against rabbit and mouse are diluted at 1:5000, and the membranes were incubated with secondary antibodies for 1 h and visualized by chemiluminescence.

### Cell transfection

Both ccRCC cells A498 and 786O cells line were transfected with miR-187-3p mimics (Cat. No. miR11272492907-1-5, RiboBio, Guangzhou, China), miR-187-3p inhibitor (Cat. No. miR20000262-1-5, RiboBio), negative control (NC, RiboBio) and LRFN1 overexpression plasmid using Lipofectamine™ 3000 reagent (Cat. No. L3000001, Invitrogen) according to the manufacturer’s instructions. Both A-498 and 786-O cells were transfected with 50 nM miRNAs, 3 µmol/l siRNAs, and 1 µg/µl overexpression plasmid (pcDNA3.1-LRFN1 from Shanghai GeneChem Co., Ltd.) using Lipofectamine™ 3000 reagent (Cat. No. L3000001, Invitrogen) according to the protocols. Cells were harvested for further investigations after transfection for 24 h.

### Cell viability assay

A498 and 786O cells line were seeded into 96-well plates at a density of 4 × 10^3^ cells/well, and cultured in a 5% CO_2_ incubator at 37 degrees Celsius for 24 h, 48 h, 72 h, and 96 h, respectively. Then, 10 μl CCK8 (MedChemExpress, HY-K0301-500 T) solution was added to each well, and the cells were cultured for 30 min–4 h according to the manufacturer’s instructions. The OD value of the medium was detected using a spectrophotometer at 450-nm wave length.

### Transwell cell invasion assay

Transwell invasion assays were performed using a 24-well transwell chamber (Greiner bio-one, Switzerland). First, Matrigel (BD Bioscience, USA) was applied to the upper ventricular surface of the Transwell chamber basement membrane, and then 2 × 10^5^ A498 and 786O cells were suspended in 0.2 ml of serum-free medium and added to the insert. The lower compartment was supplemented with 0.5 ml of medium containing 10% FBS as a chemical attractant. After incubating for 48 h at 37 °C, the cells on the upper surface of the membrane were carefully removed with a cotton swab, and the cells on the lower surface were continuously fixed with 100% methanol, and then stained with 0.5% crystal violet. Three random fields of magnification of 200X were selected for each insert, and the number of cells was counted under an optical microscope (Olympus, Japan).

### Cell apoptosis assays

Apoptosis detection assay was performed using Annexin V-FITC Apoptosis Detection Kits (BD, USA) using a FACS analyzer (BD, USA) in accordance with the manufacturer’s experiment procedures. After A498 and 786O cells were collected and washed in PBS for three times, 500 μl cell suspension, 5 μl Annexin V-FITC, and 5 μl propidium iodide (PI) solution were resuspended in each collection tube.

### Cell cycle assay

Flow cytometry was performed to measure the effect of miR-187-3p inhibitor and mimics interference on cell cycle distribution of A498 and 786O cells in comparison with NC group. After A498 and 786O cells were collected and washed in PBS for three times, and disposed using cell cycle assay kit (Nanjing KeyGen Biotech), the percentage of the cells number of each cell cycle phase (G0/G1, S, G2M) was detected using a FACS analyzer (BD, USA).

### Luciferase reporter assay

The online biological database TargetScan (http://www.targetscan.org/) was used to predict the targets of miRNAs. To investigate whether miR-187-3p could interact with the 3′-UTRs of LRFN1, wild-type (WT) 3′-UTR of LRFN1 predicted to interact with miR-187-3p or the mutant (MUT) LRFN1 3′-UTR was amplified. Then, the WT 3′-UTR of LRFN1 or MUT 3′-UTR of LRFN1, and miR-187-3p mimic were co-transfected into A498 and 786O cells by lipofectamine 8000 according to the manufacturer’s instructions. Forty-eight hours after co-transfection, the cells were lysed and assayed using Dual-Luciferase^®^ Reporter Assay Kit (Promega, Madison, WI, USA) based on the manufacturer’s instructions. The primers of WT 3′-UTR or MUT 3′-UTR of LRFN1 used as follows. WT 3′-UTR: forward, 5′‐TGG TCC GTC TGG ACA TGA C‐3′, c, 5′‐CAG GTC TCT AAG TCG TCC TCG‐3′; MUT 3′-UTR: forward, 5′-TAT CTA AGC AAG TCG CCT C-3′, reverse, 5′-AGG CGT TAC ATA TGT GAC-3′.

### Extracellular acidification assays

To confirm the role of miR-187-3p and LRNF1 in aerobic glycolysis, we performed glycolysis measurements and assessed extracellular acidification (ECAR) rate using the Seahorse XFe96 Extracellular Flux Analyzer (Seahorse Bioscience, North Billerica, USA). ECAR assay were examined using the Seahorse XF Glycolysis Stress Test Kit (Agilent Technologies, Wilmington, DE, USA) and the Seahorse XF Cell Mito Stress Test Kit (Agilent Technologies), according to the manufacturer's protocols and our previous reports [34]. Briefly, 1 × 10^3^ cells/well were seeded into a 24-well plate with medium containing 2 mM glutamine. Then, cells were incubated in a non-CO2 37 °C atmosphere for 1 h prior to the assay. Glucose (10 mM), oligomycin (1 μM), and glycolytic inhibitor 2-DG (100 mM) were injected sequentially to assess the ECAR with 3 repeated tests after each injection. Each point represents the average of three independent samples.

### Xenograft mouse model

For in vivo analysis, 6-week-old male nude mice were anesthetized and A498 cells (5 × 10^6^) premixed with Matrigel at 1: 2 were subcutaneously injected into the right rear back region. Mice were randomized into three groups (n = 6 each) and treated with DMSO (control), LRFN1 overexpression plasmid, LRFN1 plus mimics when tumors reached 100 mm^3^. The tumor size and volume were calculated every week. Tumor volume was monitored as: volume = length × (width)^2^/2. The mice were euthanized 42 days after tumor-cell injection, and tumors were dissected for immunohistochemistry analysis. We declared that the maximal tumor size was less than 15 mm in diameter in any dimension in mice. All animal testing procedures comply with the Ethics and Guidelines for Animal Research, and has been approved by Institutional Animal Care and Use Committee of FUSCC (Permit number: FUSCC-IACUC-S2022-0066) at which the experiments were conducted. This study follows the legal requirements or guidelines for the care and use of animals in China, and the International Union for Conservation of Nature (IUCN) Policy Statement on Research Involving Species at Risk of Extinction.

### Immunohistochemistry (IHC) staining analysis

Immunohistochemical analysis (IHC) was implemented with Ki-67 Polyclonal Antibody (No. 14-5698-82, Invitrogen), LRFN1 Polyclonal Antibody (No. PA5-20698, Invitrogen) and anti-PD-L1 (No. 14-5983-82, Invitrogen) at 1/500 dilution. On the basis of the integration of IHC staining degree, two experienced and independent pathologists/clinician evaluated the overall IHC score (from 0 to 12), defining a negative staining of 0–3 and a positive staining of 4–12 for each tissue, as previously described [35].

### Immune infiltration analysis

By obtaining a variety of immune infiltrating lymphocytes with different degrees of infiltration using CIBERSORT algorithm (https://cibersort.stanford.edu). To investigate the association between LRFN1 and tumor immune microenvironment of ccRCC, the abundance of immune cell infiltrations was measured using Tumor IMmune Estimation Resource (TIMER, https://cistrome.shinyapps.io/timer/). Additionally, TISIDB (http://cis.hku.hk/TISIDB/), an integrated repository portal for tumor-immune system interactions, was also implemented in investigation of ccRCC and immune system interaction based integrated multiple heterogeneous data types. The differentially infiltrated lymphocytes were evaluated using the Wilcoxon rank sum test. The Tumor Immune Dysfunction and Exclusion (TIDE) test was used to detect tumor heterogeneity between the two groups.

### Survival analysis

Survival analysis was performed using transcriptional expression of miR-187-3p and follow-up information based on the Cancer Genome Atlas (TCGA), Clinical Proteomic Tumor Analysis Consortium (CPTAC) and International Cancer Genome Consortium (ICGC, RECA-EU) databases in Kaplan–Meier methods. Progression-free survival (PFS) and overall survival (OS) were considered as the primary and secondary endpoint for patients with ccRCC. The follow-up duration was analyzed by Kaplan–Meier method with 95% confidence intervals (95% CI) and log-rank test in separate curves. To further explore significant independent variables of ccRCC, univariate and multivariate analysis were performed with Cox logistic regression models to find independent variables. X-tile software was utilized to take the cut-off value.

### Statistical analysis

In general, most data represent the mean ± standard deviation (SD) and are representative of three independent experiments. Statistical analysis was conducted using GraphPad Prism 8.0 software (GraphPad) and the R package (V. 3.6.1). All measurement data are shown as the mean ± SD. Differences in measurement data between groups were analyzed using a two-tailed Student’s t test. Kaplan–Meier survival curves with log-rank tests were evaluated to analyze the time to progression and survival. All hypothetical tests were two-sided and *P*-values less than 0.05 were considered significant in all tests.

## Results

### MiR-187-3p expression is decreased in ccRCC tissues and cells, and correlates with poor prognosis of ccRCC patients

**Fig. 1:**
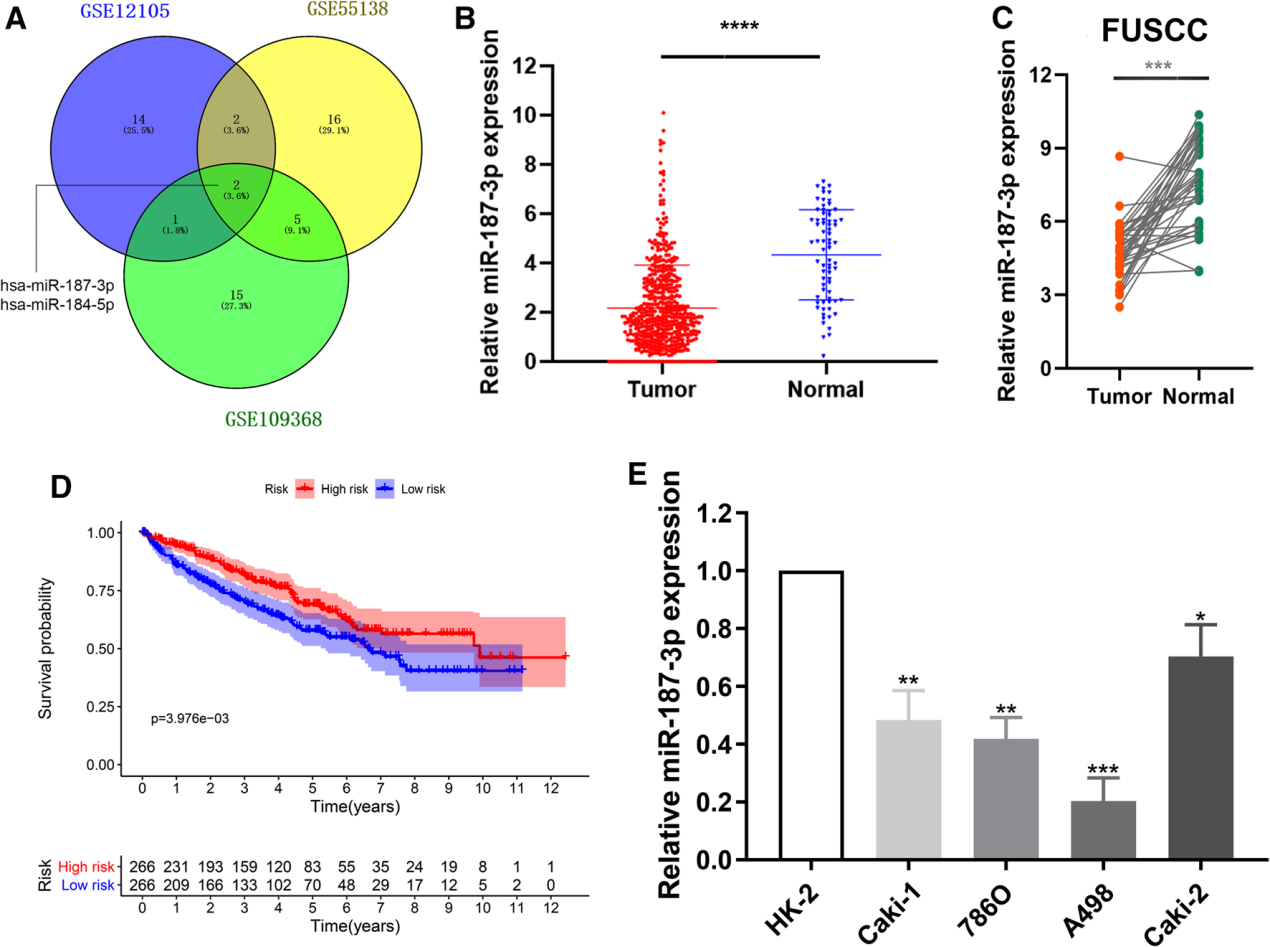
Screening and identification of miR-187-3p for ccRCC from multiply datasets. **A** The overlap among the three datasets in Venn diagram. **B** Relative miR-187-3p expression level in ccRCC and normal tissues base on TCGA cohort. **C** Relative miR-187-3p expression level in ccRCC and normal tissues based on FUSCC cohorts. **D** Survival plots of miR-187-3p expression for ccRCC patients. **E** The relative miRNA expressions of miR-187-3p in four ccRCC cell lines and a normal cell line HK-2. *P < 0.05; **P < 0.01; ***P < 0.001; ****P < 0.0001

After being normalized and censored of the miRNA microarray information, the DEmiRNAs were determined to be significant based on the analysis and the statistical parameters of the data processing steps (Supplementary Table 1). The overlap among the three datasets included two significant microRNA hsa-miR-187-3p and hsa-miR-184-5p, displayed in Venn diagram (Fig. 1A). Then, a total of 533 ccRCC tissues and 72 normal kidney tissues with available transcriptional profiles were enrolled in the present study. We found that relative miR-187-3p expression level was significantly decreased in ccRCC tissues compared with normal tissues (*P* < 0.001, Fig. 1B) based on patients with ccRCC from TCGA database. As shown in Figure S1A–C, we found that miR-184-5p could not significantly differ survival outcomes for patients with ccRCC from TCGA database in the overall survival analysis (*P* = 0.62) and the subgroup survival analyses (*P* = 0.62, *P* = 0.97, respectively) using Kaplan–Meier methods. Therefore, we selected miR-187-3p as the hub miRNA that might modulate tumorigenesis of ccRCC for further exploration.

In addition, we evaluated the expression of miR-187-3p in tumor tissues and adjacent normal tissues from FUSCC, a real-world validation cohort. Consistently, miR-187-3p expression level is significantly decreased in ccRCC tissues compared with normal tissues based on FUSCC cohort (Fig. 1C). To explore the potential prognostic value of miR-187-3p for ccRCC patients, we performed overall survival analysis. As shown in Fig. 1D, decreased miR-187-3p expression was significantly associated with poorer prognosis for ccRCC patients (*P* = 0.004). Subsequently, we detected the miR-187-3p expression level in ccRCC and normal human kidney cell line HK-2. As illustrated in Fig. 1E, compared with normal renal cell lines, the expression level of miR-187-3p was significantly reduced in A498 cells and 786O cells, which thus was selected for further subsequent experiments.

### Down-regulation of miR-187-3p promotes proliferation of A498 and 786O cells

**Fig. 2:**
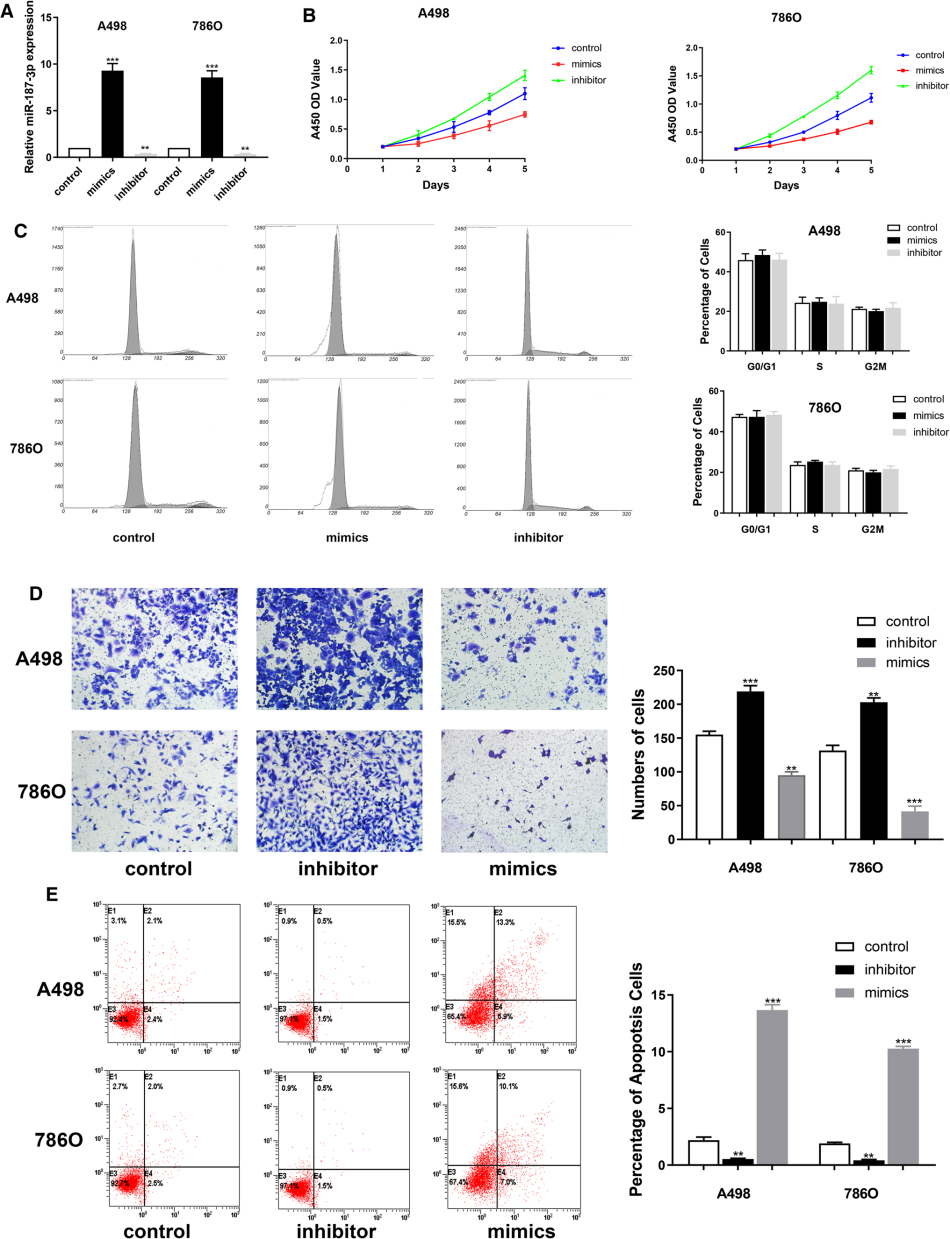
MiR-187-3p suppressed ccRCC cells growth, migration and promotes apoptosis. **A** The expression level of miR-187-3p in A498 and 786O cells after transfection with mimics, inhibitor or negative control was detected using qRT-PCT. **B** CCK-8 assay was performed to determine the proliferation of A498 and 786O cells overexpressing or down-expressing miR-187-3p. **C** Flow cytometry was performed to determine the cell cycle of A498 and 786O cells overexpressing or down-expressing miR-187-3p. **D** Transwell assay was performed to determine the migration ability of A498 and 786O cells overexpressing or down-expressing miR-187-3p. **E** Annexin V-FITC Apoptosis Detection Kits was performed to determine the cell apoptosis of A498 and 786O cells overexpressing or down-expressing miR-187-3p compared to the negative control group using a FACS analyzer. **P < 0.01; ***P < 0.001

Next, we explored whether miR-187-3p could modulate the proliferation ability of ccRCC cells. We investigated the expression level of miR-187-3p in A498 and 786O cells after transfection with mimics, inhibitor or negative control. The results demonstrated that the expression level of miR-187-3p was significantly increased in the miR-187-3p mimic-transfecting group cells (*P* < 0.001), but decreased in the miR-187-3p inhibitor-transfecting group in A498 and 786O cells (*P* < 0.01) (Fig. 2A). To detect the potential function of miR-187-3p, we assessed cell proliferation using a CCK-8 assay after transfecting miR-187-3p mimics or inhibitor into A498 and 786O cells. After being up-regulated of miR-187-3p by mimics, the cell growth rate of miR-187-3p overexpression group was significantly decreased compared to the negative control group (*P* < 0.01). Conversely, the growth rate of miR-187-3p downregulated cells group was markedly increased compared to the negative control group for both A498 and 786O cells (*P* < 0.01) (Fig. 2B). To further measure the effect of miR-187-3p in cell cycle, we transfected mimic and inhibitor of miR-187-3p into A498 and 786O cells, and found no significant differences in G0/G1, S and G2/M phases compared with the negative control (Fig. 2C).

### Down-regulation of miR-187-3p promotes migration and restrains apoptosis ability in A498 and 786O cells

Migration assays were performed to assess malignant ability after transfecting miR-187-3p mimic or inhibitor into A498 and 786O cells. The results indicated that upregulation of miR-187-3p could significantly reduce the migration capacity, but cell numbers markedly increased after transfection with of miR-187-3p inhibitor, compared to the negative control group (*P* < 0.001) (Fig. 2D). As shown in Fig. 2E, after transfection with miR-187-3p inhibitor in A498 and 786O cells, we found significantly decreased apoptosis cells compared to negative control cell groups measured by propidium iodide (PI) and FITC—Annexin V fluorescence. When transfecting miR-187-3p mimics, A498 and 786O cells markedly increased compared with the negative control group (*P* < 0.01). Overall, down-regulation of miR-187-3p promotes proliferation, migration and restrains apoptosis ability of A498 and 786O cells.

### MiR-187-3p directly targets LRFN1-3′-UTR and modulates LRFN1 expression

**Fig. 3:**
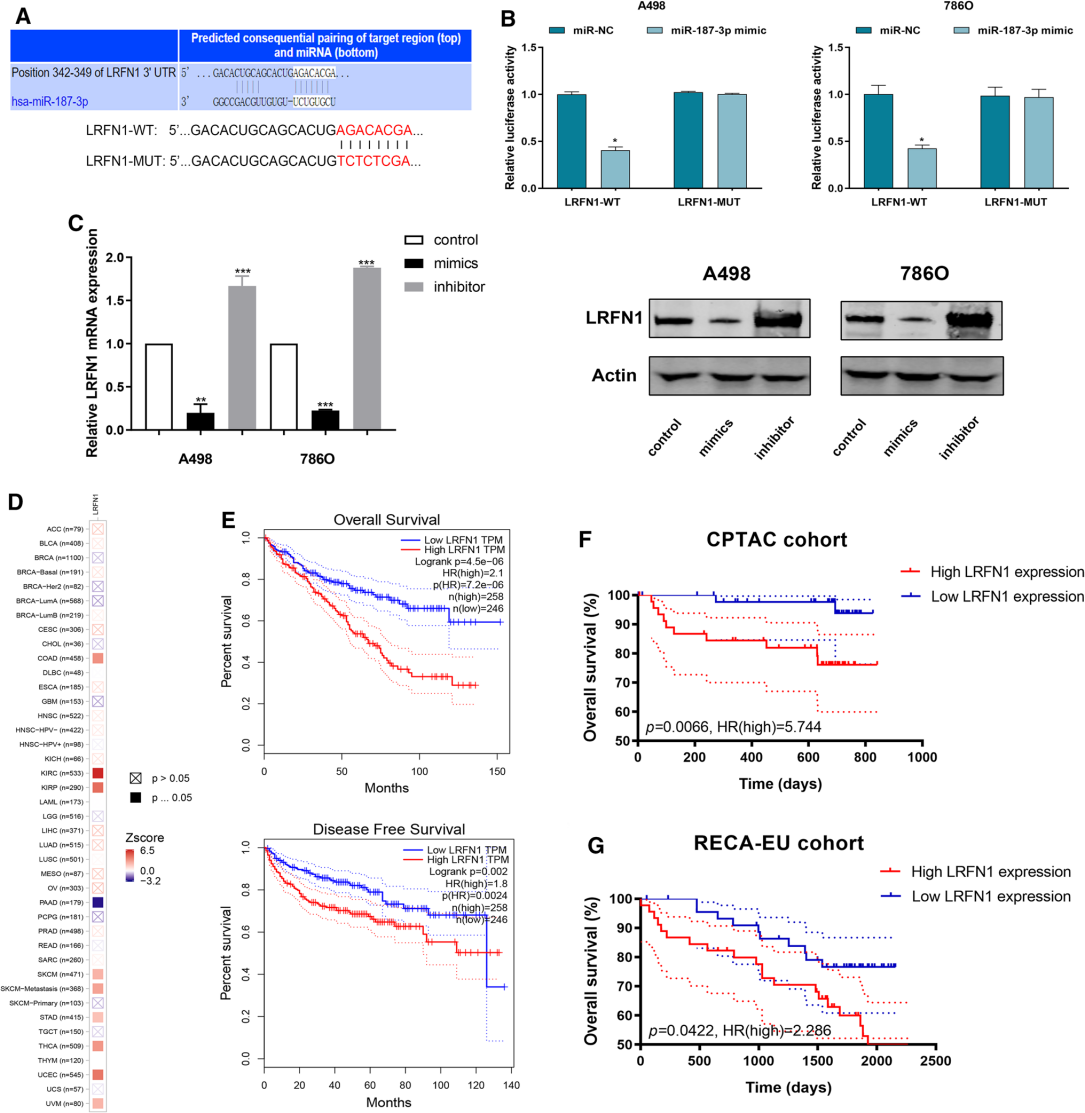
MiR-187-3p directly targets LRFN1-3′-UTR and modulates the prognostic LRFN1 expression. **A** A putative binding site of miR-187-3p in the 3′-UTR of LRFN1. **B** Luciferase assay was performed to investigate whether miR-187-3p binds to a putative binding site in the 3′-UTR of LRFN1. **C** RT-qPCR and Western Blot were performed to assess the transcriptional and protein expression level alteration of LRFN1. **D** Prognostic implication of LRFN1 in 30 different tumors from TCGA database using log rank test [− log10(P-value)]. **E** Kaplan–Meier survival plots were used to evaluate predictive value of LRFN1 expression in OS and PFS for 504 patients with ccRCC from TCGA cohort. **F** Kaplan–Meier survival plots suggested prognostic role of LRFN1 expression for ccRCC patients from CPTAC cohort. **G** Kaplan–Meier survival plots suggested prognostic role of LRFN1 expression for ccRCC patients from RECA-EU cohorts. *P < 0.05; ***P < 0.001

To reveal the underlying molecular mechanism and biological function of miR-187-3p in ccRCC cells, webtools TargetScan Human 7.2 was used to search for downstream targets of miR-187-3p. Eventually, LRFN1 was recognized as a potential downstream target of miR-187-3p. As shown in Fig. 3A, the 3′-UTR of LRFN1 contains a putative binding site for miR-187-3p. Then, we performed a Luciferase assay to investigate whether miR-187-3p binds to a putative binding site in the 3′-UTR of LRFN1. Overexpression of miR-187-3p decreased the luciferase activity of WT LRFN1 3′-UTR (*P* < 0.05), while miR-187-3p overexpression did not have marked influence on the Luciferase activity of MUT LRFN1 3′-UTR (Fig. 3B). Next, we detected mRNA and protein expression alteration of LRFN1 after transfecting miR-187-3p mimic or inhibitor into A498 and 786O cells using western blotting and qRT-PCR assays. Significantly decreased and increased expression level was respectively observed in mimic and inhibitor groups, suggesting a direct effect of miR-187-3p on LRFN1 in A498 and 786O cells (Fig. 3C). Overall, these results showed that miR-187-3p negatively modulates LRFN1 protein expression by directly binding to the 3′-UTR of LRFN1 in human ccRCC cells.

### Expression of miR-187-3p and LRFN1 are both prominently independent prognostic signatures for patients with ccRCC

To investigate the relationships between LRFN1 mRNA expression and prognosis of ccRCC patients, we enrolled patients with ccRCC from TCGA, CPTAC and ICGC databases. Prognostic implication of LRFN1 expression among pan-cancers from TCGA database using log rank test [− log10(P-value)] was shown in Fig. 3D. LRFN1 expression showed the strongest association with prognosis of ccRCC patients (Zscore = 6.484), and significantly predicts prognosis for patients with COAD, KIRP, PRAD, SKCM, STAD and THCA. In addition, after identifying differential LRFN1 expression using the median cutoff value, we found that high LRFN1 expression markedly correlated with poor prognosis (*P* = 4.5E−06) and aggressive progression (*P* = 0.002) in patients with ccRCC from TCGA cohort (Fig. 3E). Then, survival analysis suggested that higher LRFN1 expression was prominently associated with poor OS for ccRCC patients from CPTAC (*P* = 0.0066, HR = 5.744; Fig. 3F) and RECA-EU cohorts (*P* = 0.0422, HR = 2.286; Fig. 3G). Overall, elevated LRFN1 mRNA expression was significantly associated with advanced clinicopathological parameters and poor prognosis in ccRCC patients.

Subsequently, to explore whether miR-187-3p and LRFN1 are independent indicators for prognosis of ccRCC, we implemented univariate and multivariate Cox regression analysis. The univariate Cox analysis suggested that decreased miR-187-3p and elevated LRFN1 expression significantly predict poor prognosis for patients with ccRCC (*P* = 0.007, *P* < 0.001, respectively; Figure S2A, B). In the forest plot, multivariate Cox analysis indicated that both miR-187-3p (*P* = 0.026, HR = 0.824) and LRFN1 (*P* = 0.001, HR = 1.610) expression could serve as potential promising biomarkers for prognosis of ccRCC (Figure S2C, D). In TCGA cohort, increased LRFN1 expression also significantly correlated with high tumor grade and individual clinical cancer stage (*P* < 0.001). The highest LRFN1 expression was observed in grade 4 or stage 4 group, respectively (Figure S3A, B). Furthermore, expression of LRFN1 was significantly higher in BCa samples with nodal metastasis compared with those without lymph node metastasis (*P* < 0.05, Figure S3C).

### LRFN1 rescued cells proliferation and migration capacities after miR-187-3p mimic transfection

**Fig. 4:**
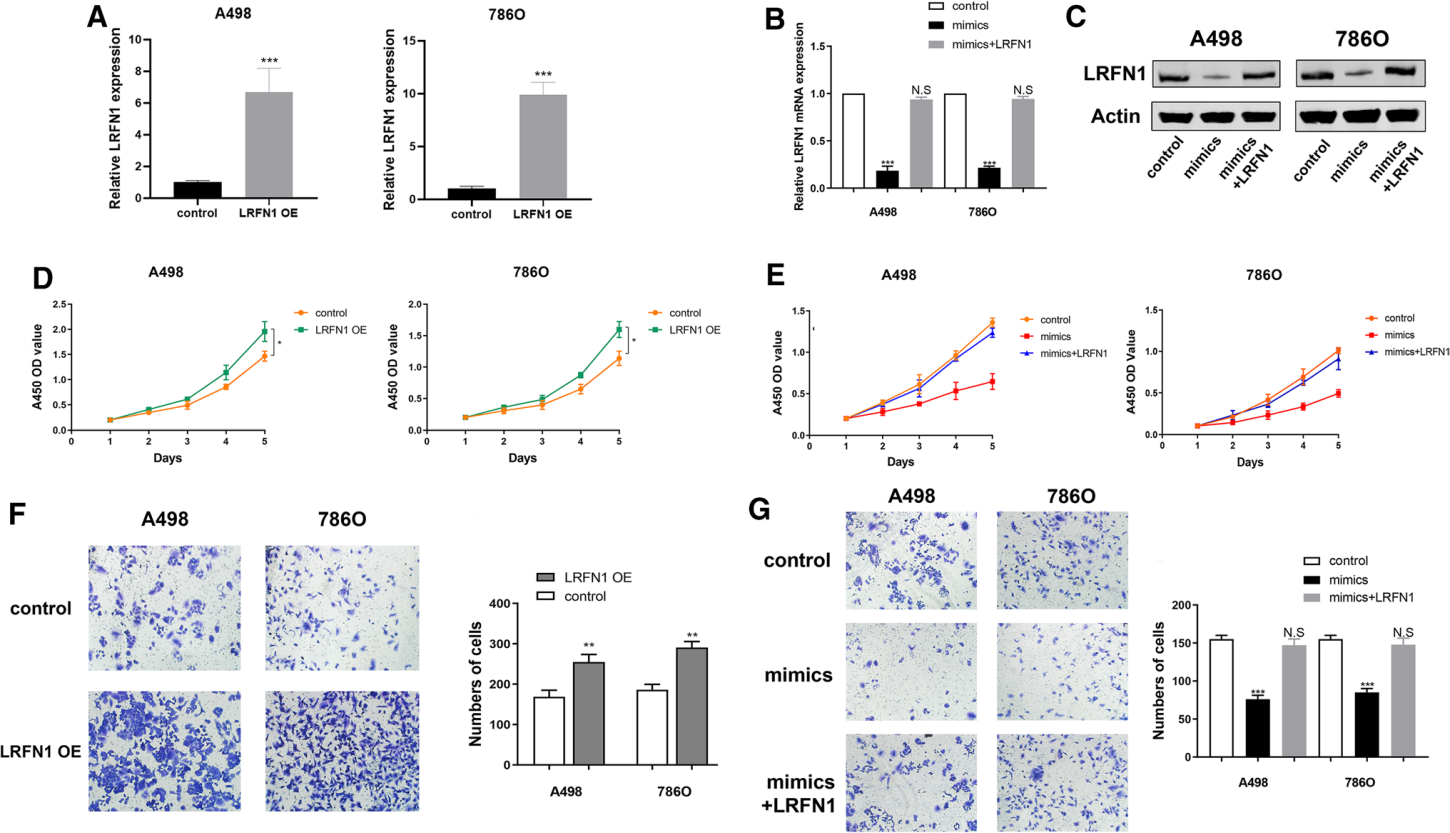
LRFN1 rescues ccRCC cells proliferation, migration after miR-187-3p mimic transfection. **A** RT-qPCR was performed to assess LRFN1 mRNA expression in A498 and 786O cells. **B**, **C** RT-qPCR and Western Blot was performed to assess LRFN1 mRNA and protein expression in A498 and 786O cells. **D**, **E** CCK-8 assay was performed to measure cell proliferation in A498 and 786O cells. **F**, **G** Transwell assay was performed to determine the migration ability of A498 and 786O cells overexpressing LRFN1 and mimics, mimics + LRFN1 groups. Statistical significance was calculated using Student’s t test *P < 0.05; **P < 0.01; ***P < 0.001

Relative LRFN1 mRNA and protein expression level alteration was detected after transfecting miR-187-3p mimic or transfecting miR-187-3p mimic combined with LRFN1 compared with negative control. It suggested a significant reduction of LRFN1 mRNA and protein expression level after transfecting mimics, while relative expression was rescued to normal when exposed to LRFN1 compared to the negative control group (Fig. 4A). Additionally, to explore the effect of LRFN1 on cell proliferation and invasion, LRFN1 was transfected into A498 and 786O cells respectively, and then the changes in LRFN1 mRNA expression levels were quantified. As shown in Fig. 4B, C, the expression of LRFN1 was significantly increased.

Subsequently, to explore whether miR-187-3p inhibits proliferation of A498 and 786O cells by directly targeting LRFN1, we assessed CCK-8 assay after transfecting miR-187-3p mimics or miR-187-3p mimics combined with LRFN1 into A498 and 786O cells, compared with negative control. It suggested that cell proliferation was significantly decreased in miR-187-3p upregulation group, while cell numbers were rescued to the similar as the negative control group (Fig. 4D); proliferation was significantly enhanced after LRFN1 was overexpressed in ccRCC cells (Fig. 4E).

In addition, results of invasion and migration assay showed that cell migration vitality was significantly decreased in miR-187-3p mimic group, while cell numbers were rescued to normal as the negative control group in A498 and 786O cells (Fig. 4F). Similarly, Transwell analysis showed that the invasion of ccRCC cells was significantly enhanced after LRFN1 overexpression (Fig. 4G). Therefore, these data indicated that miR-187-3p suppresses the malignant phenotype of ccRCC cells by targeting LRFN1.

### Identification of differential expressed genes and functional annotations of LRFN1 in silico

**Fig. 5:**
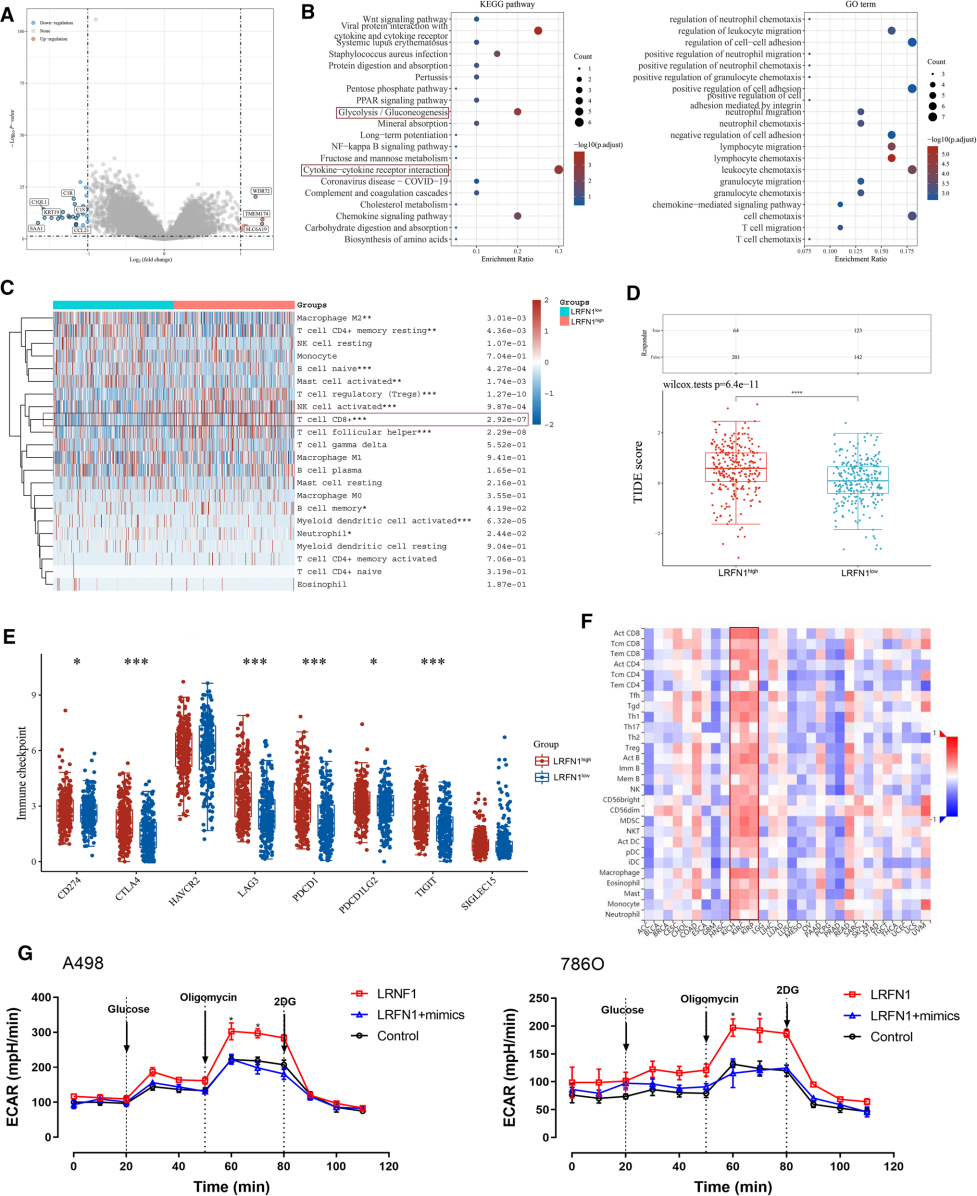
LRFN1 promotes immune-infiltrated TIME and aerobic glycolytic effects of ccRCC. **A** Limma R package was performed to identify the significant DEGs according to LRFN1 expression. **B** KEGG and GO functional enrichment analysis suggested that the LRFN1-related DEGs prominently involved in the hallmarks associated with tumorigenesis and progression. **C** CIBERSORT algorithm was performed to characterize immune cell composition of complex tissues from their gene expression profiles of ccRCC based on transcriptome data from TCGA database. **D** TIDE algorithm was performed to measure intratumoral heterogeneity of ccRCC with Student’s t test. **E** Expression of immune checkpoints were assessed using unpaired t test. **F** Association between LRNF1 expression and the infiltration of tumor immune-infiltrating lymphocytes in pan-cancer was assessed using Spearson’s correlation analysis. **G** ECAR of ccRCC cells were analyzed by a Seahorse XFe 96 Extracellular Flux Analyzer. All results are representative of at least three independent experiments. Asterisk represents significant differential ECAR value between LRNF1 overexpression group and negative control group, as well as between LRNF1 overexpression group and LRFN1 + mimics group using unpaired t test (*P* < 0.05). *P < 0.05; ***P < 0.001; ****P < 0.0001

To investigate the potential functions of LRFN1 in the progression of ccRCC, we screened and identified the significantly differential expressed genes (DEGs) according to LRFN1 expression, and found the significantly up-regulated genes, including WDR32, TMEM174 and SLC6A19; down-regulated genes, including C1R, C1S, CCL21, C1QL1, KRT19 and SAA1 (Fig. 5A). Subsequently, KEGG enrichment analysis suggested that the LRFN1-related DEGs prominently involved in the hallmarks associated with tumorigenesis and progression, specifically mediating the tumor metabolism Glycolysis/Gluconeogenesis and the tumor immune microenvironment (TIME) cytokine-cytokine receptor interaction. GO functional enrichment analysis further revealed that LRNF1 might participate in the regulation of leukocyte, neutrophil, lymphocyte, granulocyte and T cells, suggesting that LRNF1 is most likely involved in the remodeling of TIME in ccRCC (Fig. 5B).

### LRFN1 promotes immune-infiltrated TIME and glycolytic effects of ccRCC

Next, based on CIBERSORT algorithm, we characterized immune cell composition of complex tissues from their gene expression profiles of ccRCC based on TCGA. As shown in Fig. 5C, we found significantly enriched CD8^+^ T cells and Treg infiltration, while decreased B cells naive and myeloid dentritic cell activated in the group of high LRNF1 expression. Next, using TIDE algorithm, we found significantly higher intra-tumoral heterogeneity for ccRCC patients with higher LRNF1 expression, which normally results in treatment tolerance, disease progression, and poor prognosis (Fig. 5D). Besides, for patients with higher LRNF1 expression, the expression of most immune checkpoints are significantly increased, including CD274, CTLA-4, LAG-3, PD-1, PD-L2, and TIGIT (Fig. 5E). Interestingly, after reviewing the association between LRFN1 expression and immune cells infiltration levels in pan-cancer, we found that LRFN1 was highly consistent with the infiltration of tumor immune-infiltrating lymphocytes, but this finding was only present in RCC (including three different subtypes) instead of other cancers (Fig. 5F). RCC is a type of cancer with highly active aerobic glycolytic metabolism. According to the results of KEGG analysis, we further found that up-regulation of LRNF1 significantly promotes ECAR and mimics of miR-187-3p significantly rescued the glycolysis effect of ccRCC cells (Fig. 5G). The findings suggested that the ECAR value in LRNF1 overexpression group was significantly up-regulated within the interval between the addition of Oligomycin and 2-DG compared with either negative control group or LRFN1 + mimics group (*P* < 0.05). Taken together, these findings revealed that LRNF1 might modulate aerobic glycolysis activity of ccRCC, and thus promoting the immune-infiltrating TIME in ccRCC.

### Overexpression of LRFN1 promotes ccRCC growth and immune-infiltrating TIME in a xenograft model

**Fig. 6:**
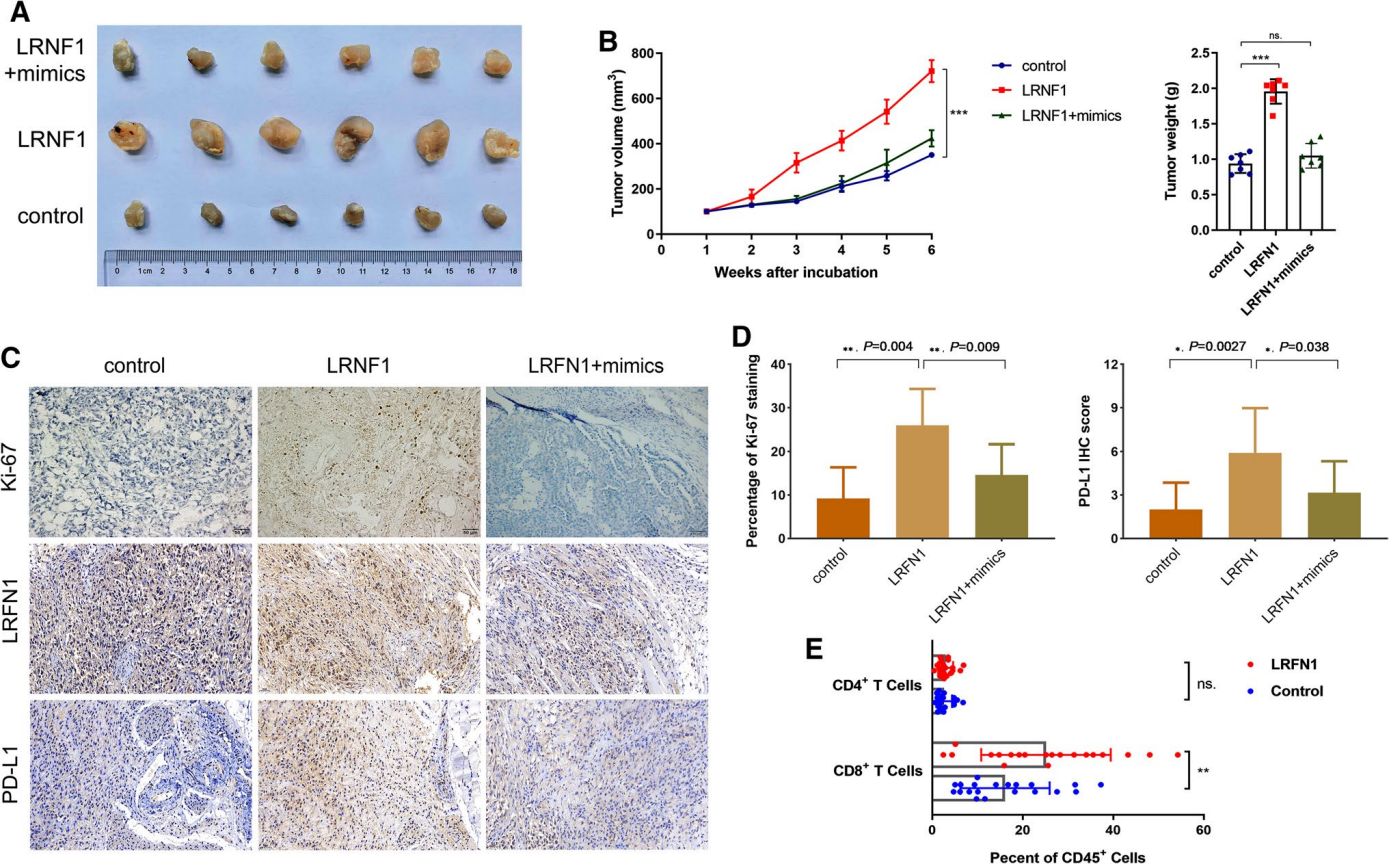
LRFN1 promotes ccRCC growth and immune-infiltrating TIME on a xenograft model. **A** The representative images of three groups (control, LRFN1 overexpression plasmid, LRFN1 plus miR-187-3p mimics) on the xenograft model. **B** miR-187-3p rescues LRFN1 promoted A498 cell volume and weight in vivo. **C**, **D** The IHC staining was performed to assess Ki-67 staining, LRFN1 expression and PD-L1 expression of three groups. **E** The Flow cytometry assay was performed to reveal active CD4^+^ and CD8^+^ T cells in tumor tissues between high and low LRFN1 expression groups. *P < 0.05; **P < 0.01; ***P < 0.001

To further confirm the in vitro findings, we observed the biological roles of miR-187-3p and LRFN1 in vivo. The representative images of three groups (control, LRFN1 overexpression plasmid, LRFN1 plus miR-187-3p mimics) are shown in Fig. 6A. LRFN1 overexpression in A498 cells increased the tumour volume 1.79-fold relative to that of its control and increased the tumour weight 2.16-fold relative to its control (Fig. 6B). The results demonstrated that after implantation of LRFN1overexpressing cells, tumor xenografts grew significantly faster than those in the control group, while the growth could be rescued by miR-187-3p. Additionally, the IHC staining of Ki-67, LRFN1 and PD-L1 were performed of three groups from xenograft model. The results revealed that the percentage of Ki-67 staining and IHC score of PD-L1 was significantly higher in LRFN1 group compared with negative control (*P* < 0.05, Fig. 6C, D). The Flow cytometry results showed higher active CD8^+^ T cells in the LRNF1^high^ group (Fig. 6E). Overall, the up-regulation of LRFN1 promotes ccRCC growth and immune-infiltrating TIME in vivo.

## Discussion

Extensive research finds that many miRNAs play key roles in various cancers [36]. Accumulating evidence shows that miRNAs are promising biomarkers and therapeutic targets in ccRCC [37]. MicroRNAs in tissues and serum have been widespread considered as effective tools for early detection treatment targets of tumors. There existing many promising and effective downstream targets of miRNAs in modulating tumorigenesis [38, 39]. In this study, we identified that LRFN1 was a novel downstream target of miR-187-3p in ccRCC based on the bioinformatics analyses and experimental data from luciferase assay, qRT-PCR and western blot assays. Our data demonstrated that inhibiting LRFN1 was critical for miR-187-3p exerting its suppressive effects on the growth of human ccRCC cells and the xenograft models. Interestingly, as one significant member of leucine-rich repeat and fibronectin III domain-containing family, LRFN1 is considered as a novel signature of neuronal transmembrane proteins [40], while its aggressive role in ccRCC was merely mentioned. Therefore, in this study, we not only detected differential expression of miR-187-3p and its prognostic implications in human ccRCC, but also performed CCK-8, migration, cell apoptosis and cell cycle assays to explore the biological role of miR-187-3p/LRFN1 axis in modulating malignancy and TME of ccRCC. Meanwhile, up-regulating miR-187-3p by transfection with miR-187-3p mimics could significantly restrain A498 and 786O cell proliferation, migration capabilities. Among large-scale ccRCC samples from multiply cohorts, we also proved that expression miR-187-3p and LRFN1 were both prominently independent prognostic signatures for patients with ccRCC.

Tumor microenvironment (TME) refers to the local pathological environment of tumor occurrence and development [41]. With the deepening of research, more evidence shows that the efficacy of ccRCC immunotherapy is more dependent on the activation of the TIME [42, 43]. Therefore, elucidating the molecular mechanism of immune resistance in ccRCC from different perspectives will help to deepen the understanding of the interaction between tumor cells and TME, thereby improving the overall efficacy of renal cancer treatment [44, 45]. Considering that a large number of lymphocyte infiltration led by CD8^+^ T cells and the general increase of immune checkpoint molecules are in the LRFN1^high^ group, and this phenomenon is specific in RCC, this study demonstrated that LRNF1 may affect glycolytic effects of ccRCC cells to evade immune surveillance [46], and thus mediating the immune-infiltrating TIME in ccRCC.

The rapid development of the field of immunometabolism provides a good prospect for tumor immunotherapy targeting metabolism [47–49]. Therefore, exploring the underlying mechanisms of glycolytic metabolism-driven tumor development and TAM regulation of the immune microenvironment is of great significance for preventing tumor metastasis, overcoming acquired immune resistance, and improving efficacy [50]. These novel findings allow researchers to clearly elucidate the pathological significance of the high expression of a key disease-related factor miR-187-3p in ccRCC cells, and to explore the molecular mechanism of miR-187-3p that mediates the malignant biological behavior and immune-infiltrating TIME of ccRCC cells through LRFN1. Interestingly, the Flow cytometry results showed higher active CD8^+^ T cells in the LRNF1^high^ group compared with the negative control group. These results could help to provide a new theory to the reprogram of TIME and novel insights into immune resistance mechanism. However, this study failed to supplement the flow cytometry assay to assess percentage of active CD4^+^ and CD8^+^ T cells on the LRFN1 plus mimics group, which might limit the strength of evidence.

## Conclusion

In conclusion, the present study demonstrates that decreased miR-187-3p expression contributes to inhibit proliferation, migration, apoptosis and glycolytic effects in ccRCC cells by targeting LRFN1. Our data revealed the tumor-specific and immunological role of miR-187-3p/LRFN1 axis, and highlighted the reprogram of TME and potential clinical applications in ccRCC.

## Supplementary Information

Additional file 1 Figure S1. Prognostic implications of miR-184-5p expression for patients with ccRCC from TCGA database in the A overall survival analysis and the B, C subgroup survival analyses using Kaplan–Meier methods. (TIF 1125 KB)

Additional file 2 Figure S2. Transcriptional expressions of miRNA-187-3p and LRFN1 significantly correlated with advanced clinicopathological parameters and poor survival outcomes in ccRCC patients. A, B Multivariate Cox regression analysis of miRNA-187-3p and LRFN1 predicting PFS for patients with ccRCC from TCGA database. C, D Multivariate Cox regression analysis of miRNA-187-3p and LRFN1 predicting OS for patients with ccRCC from TCGA database. (TIF 214 KB)

Additional file 3 Figure S3. LRFN1 significantly correlated with advanced clinicopathological parameters for ccRCC patients. A, B Differential LRFN1 expression with tumor grade and individual clinical cancer stage for patients with ccRCC from TCGA database using unpaired t test. C Differential LRFN1 expression with nodal metastasis status for patients with ccRCC from TCGA database using unpaired t test. *P < 0.05; **P < 0.01; ***P < 0.001; ****P < 0.0001. (TIF 124 KB)

Additional file 4 Table S1. Differential expression miRNA after screening and identifications. (XLSX 1411 KB)

## Data Availability

The datasets during and/or analyzed during the current study available from online public databases or the corresponding authors on reasonable request.

## Abbreviations

ccRCC: Clear cell renal cell carcinoma
CPTAC: Clinical Proteomic Tumor Analysis Consortium
DEGs: Differential expressed genes
DEmiRNAs: Differential expressed miRNAs
ECAR: Extracellular acidification rate
FUSCC: Fudan University Shanghai Cancer Center
GEO: Gene Expression Omnibus
ICGC: International Cancer Genome Consortium
LRFN1: Leucine rich repeat and fibronectin type III domain containing 1
OS: Overall survival
TIME: Tumor immune microenvironment
TME: Tumor microenvironment
PFS: Progression-free survival
RCC: Renal cell carcinomas
SD: Standard deviation
TCGA: The Cancer Genome Atlas

## References

[1] Siegel RL, Miller KD, Jemal A (2020). Cancer statistics, 2020. CA Cancer J Clin.

[2] Sung H (2021). Global cancer statistics 2020: GLOBOCAN estimates of incidence and mortality worldwide for 36 cancers in 185 countries. CA Cancer J Clin.

[3] Cao W (2021). Changing profiles of cancer burden worldwide and in China: a secondary analysis of the global cancer statistics 2020. Chin Med J.

[4] Zheng R (2022). Cancer incidence and mortality in China, 2016. J Natl Cancer Cent.

[5] Baldewijns MM (2008). Genetics and epigenetics of renal cell cancer. Biochim Biophys Acta.

[6] Fang Y (2021). Genetic architecture of childhood kidney and urological diseases in China. Phenomics.

[7] Sellner F, Thalhammer S, Klimpfinger M (2022). Isolated pancreatic metastases of renal cell cancer: genetics and epigenetics of an unusual tumour entity. Cancers.

[8] Linehan WM, Ricketts CJ (2019). The Cancer Genome Atlas of renal cell carcinoma: findings and clinical implications. Nat Rev Urol.

[9] Bakouny Z (2020). State of the future: translational approaches in renal cell carcinoma in the immunotherapy era. Eur Urol Focus.

[10] Zhao J (2022). Emerging regulatory mechanisms of n6-methyladenosine modification in cancer metastasis. Phenomics.

[11] Qu Y (2022). A proteogenomic analysis of clear cell renal cell carcinoma in a Chinese population. Nat Commun.

[12] Ambros V (2004). The functions of animal microRNAs. Nature.

[13] Sun K, Lai EC (2013). Adult-specific functions of animal microRNAs. Nat Rev Genet.

[14] Liu W (2022). miR-184-5p inhibits cell proliferation, invasion and predicts prognosis of clear cell renal cell carcinoma by targeting NUS1 dehydrodolichyl diphosphate synthase subunit: results from large-scale comprehensive identification and validation. J Cancer.

[15] Li D, Hao X, Song Y (2018). Identification of the key microRNAs and the miRNA-mRNA regulatory pathways in prostate cancer by bioinformatics methods. Biomed Res Int.

[16] Rodriguez-Martinez A (2019). Exosomal miRNA profile as complementary tool in the diagnostic and prediction of treatment response in localized breast cancer under neoadjuvant chemotherapy. Breast Cancer Res.

[17] Lou W (2019). Identification of potential miRNA-mRNA regulatory network contributing to pathogenesis of HBV-related HCC. J Transl Med.

[18] Nishiuchi A (2019). MicroRNA-9-5p-CDX2 axis: a useful prognostic biomarker for patients with stage II/III colorectal cancer. Cancers.

[19] Schetter AJ, Okayama H, Harris CC (2012). The role of microRNAs in colorectal cancer. Cancer J.

[20] Yang M (2019). Biosci Rep.

[21] Weng M (2017). Noncoding RNAs in the development, diagnosis, and prognosis of colorectal cancer. Transl Res.

[22] Abu-Duhier FM (2018). Clinical significance of circulatory miRNA-21 as an efficient non-invasive biomarker for the screening of lung cancer patients. Asian Pac J Cancer Prev.

[23] Shkurnikov M (2019). LAMA4-regulating miR-4274 and its host gene SORCS2 play a role in IGFBP6-dependent effects on phenotype of basal-like breast cancer. Front Mol Biosci.

[24] Bloomston M (2007). MicroRNA expression patterns to differentiate pancreatic adenocarcinoma from normal pancreas and chronic pancreatitis. JAMA.

[25] Blenkiron C (2007). MicroRNA expression profiling of human breast cancer identifies new markers of tumor subtype. Genome Biol.

[26] Nayak B (2020). Role of miRNA-182 and miRNA-187 as potential biomarkers in prostate cancer and its correlation with the staging of prostate cancer. Int Braz J Urol.

[27] Sun C (2016). MicroRNA-187-3p mitigates non-small cell lung cancer (NSCLC) development through down-regulation of BCL6. Biochem Biophys Res Commun.

[28] Maekawa S (2014). RNA sequencing: from sample preparation to analysis. Methods Mol Biol.

[29] Xu W (2021). Prognostic immunophenotyping clusters of clear cell renal cell carcinoma defined by the unique tumor immune microenvironment. Front Cell Dev Biol.

[30] Xu W (2021). Systematic genome-wide profiles reveal alternative splicing landscape and implications of splicing regulator DExD-Box Helicase 21 in aggressive progression of adrenocortical carcinoma. Phenomics.

[31] Jung M (2009). MicroRNA profiling of clear cell renal cell cancer identifies a robust signature to define renal malignancy. J Cell Mol Med.

[32] Jingushi K (2015). miR-629 targets TRIM33 to promote TGFbeta/Smad signaling and metastatic phenotypes in ccRCC. Mol Cancer Res.

[33] Gautier L (2004). affy–analysis of Affymetrix GeneChip data at the probe level. Bioinformatics.

[34] Wang J (2020). GLUT1 is an AR target contributing to tumor growth and glycolysis in castration-resistant and enzalutamide-resistant prostate cancers. Cancer Lett.

[35] Xu W (2022). Prognostic value, DNA variation and immunologic features of a tertiary lymphoid structure-related chemokine signature in clear cell renal cell carcinoma. Cancer Immunol Immunother.

[36] Quan J (2018). Tumor suppressor miR-211-5p is associated with cellular migration, proliferation and apoptosis in renal cell carcinoma. Exp Ther Med.

[37] Yang M (2019). Biosci Rep.

[38] Karreth FA, Pandolfi PP (2013). ceRNA cross-talk in cancer: when ce-bling rivalries go awry. Cancer Discov.

[39] Song YX (2017). Non-coding RNAs participate in the regulatory network of CLDN4 via ceRNA mediated miRNA evasion. Nat Commun.

[40] Morimura N (2006). Comparative analysis of structure, expression and PSD95-binding capacity of Lrfn, a novel family of neuronal transmembrane proteins. Gene.

[41] Bi K (2021). Tumor and immune reprogramming during immunotherapy in advanced renal cell carcinoma. Cancer Cell.

[42] Brown JM, Recht L, Strober S (2017). The promise of targeting macrophages in cancer therapy. Clin Cancer Res.

[43] Xu W (2021). Hexokinase 3 dysfunction promotes tumorigenesis and immune escape by upregulating monocyte/macrophage infiltration into the clear cell renal cell carcinoma microenvironment. Int J Biol Sci.

[44] Xu W (2021). Comprehensive multi-omics identification of interferon-gamma response characteristics reveals that RBCK1 regulates the immunosuppressive microenvironment of renal cell carcinoma. Front Immunol.

[45] Kasherman L (2022). Angiogenesis Inhibitors and Immunomodulation in Renal Cell Cancers: The Past, Present, and Future. Cancers.

[46] Liu Y (2021). Tumors exploit FTO-mediated regulation of glycolytic metabolism to evade immune surveillance. Cell Metab.

[47] Ganapathy-Kanniappan S (2017). Taming tumor glycolysis and potential implications for immunotherapy. Front Oncol.

[48] Colegio OR (2014). Functional polarization of tumour-associated macrophages by tumour-derived lactic acid. Nature.

[49] Xu W (2020). Fatty acid synthase correlates with prognosis-related abdominal adipose distribution and metabolic disorders of clear cell renal cell carcinoma. Front Mol Biosci.

[50] Tian X (2022). Special issue “The advance of solid tumor research in China”: Multi-omics analysis based on 1311 clear cell renal cell carcinoma samples identify a glycolysis signature associated with prognosis and treatment response. Int J Cancer.

