# Supplementary figures and images for: Deciphering the role of miR-187-3p/LRFN1 axis in modulating progression, aerobic glycolysis and immune microenvironment of clear cell renal cell carcinoma
Source: Discov Oncol. 2022 Jul 7;13:59. doi: 10.1007/s12672-022-00523-z (PMC9263027; doi:10.1007/s12672-022-00523-z)

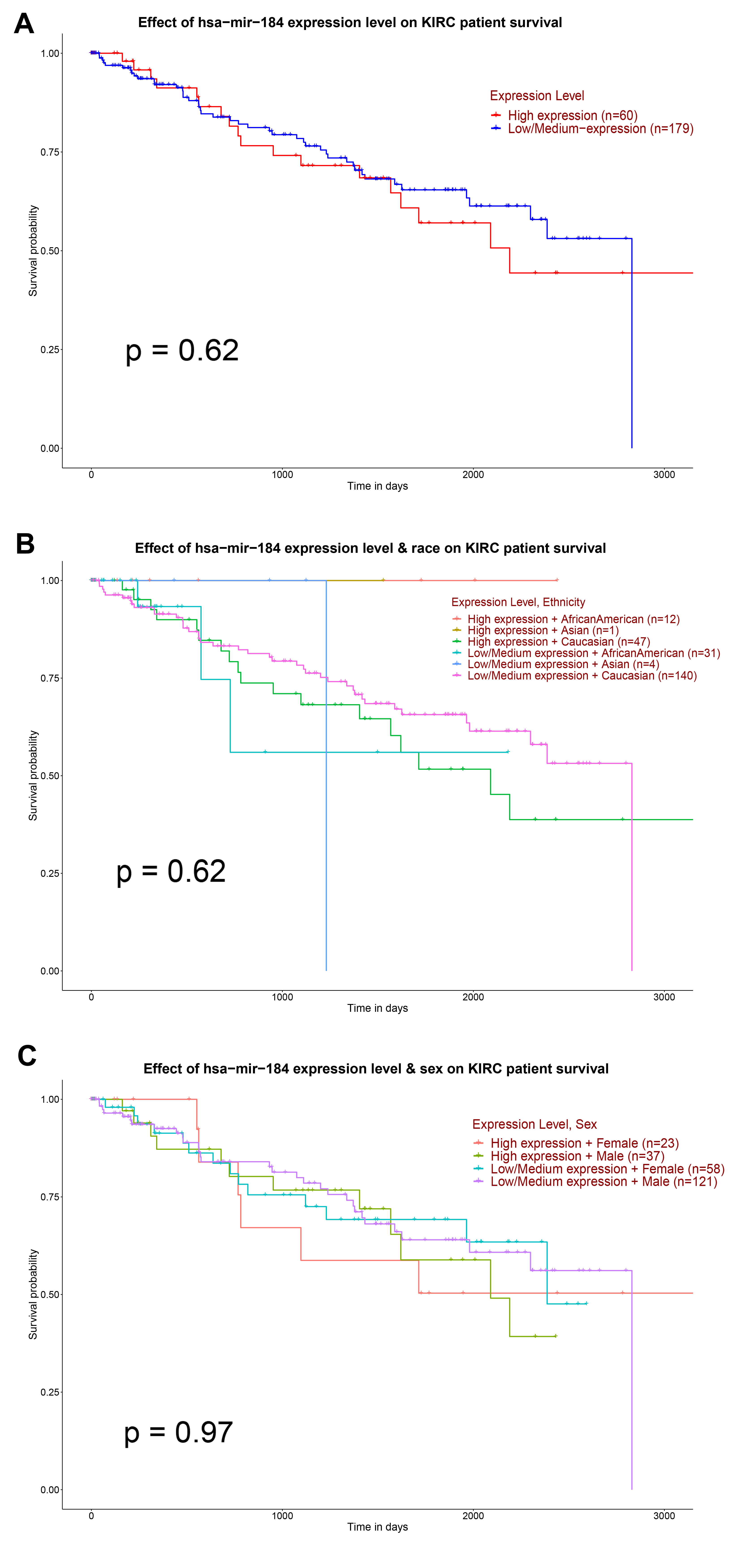

Supplement: Supplementary file 1 — Additional file 1 Figure S1. Prognostic implications of miR-184-5p expression for patients with ccRCC from TCGA database in the A overall survival analysis and the B, C subgroup survival analyses using Kaplan–Meier methods. (TIF 1125 KB) [file 12672_2022_523_MOESM1_ESM.tif]

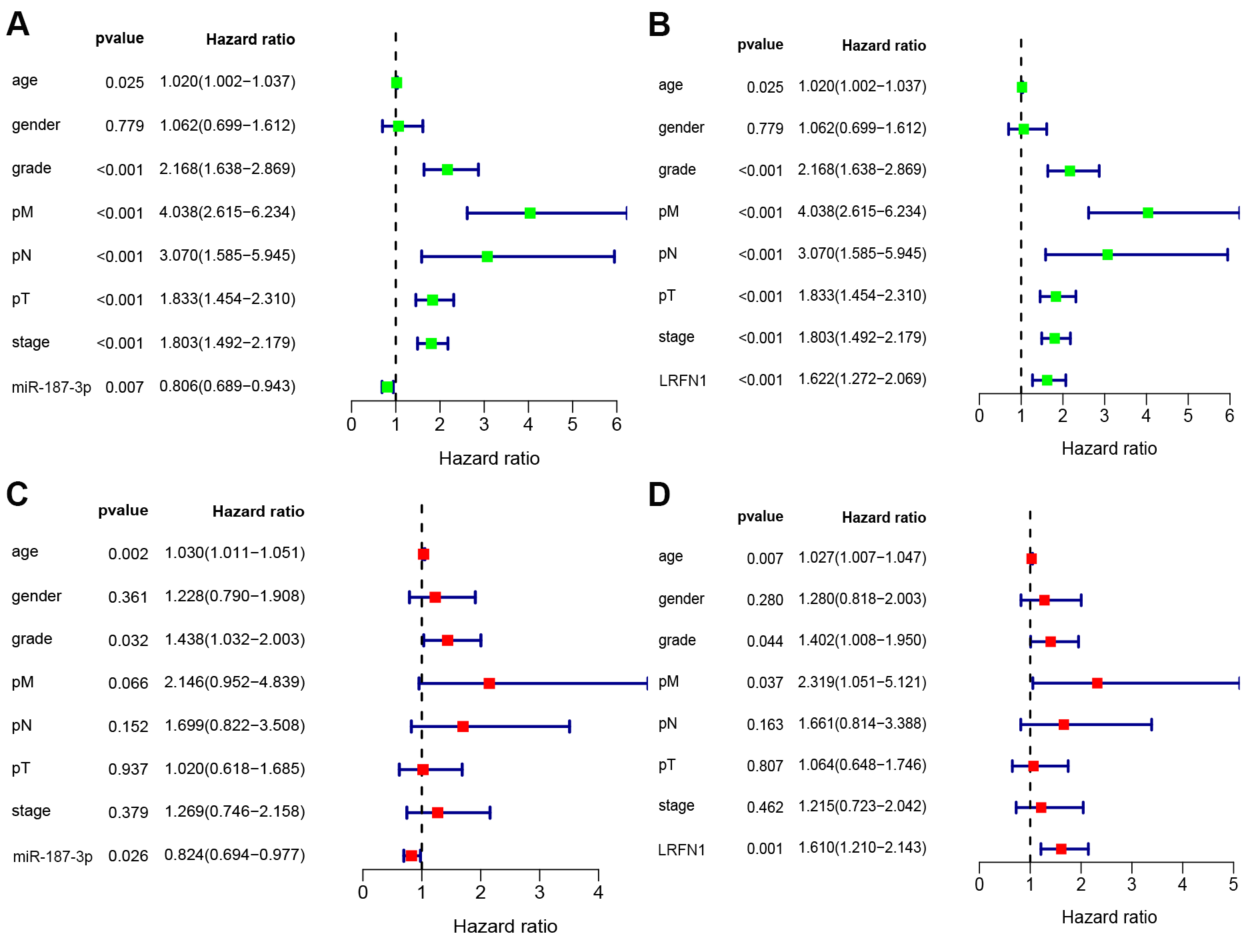

Supplement: Supplementary file 2 — Additional file 2 Figure S2. Transcriptional expressions of miRNA-187-3p and LRFN1 significantly correlated with advanced clinicopathological parameters and poor survival outcomes in ccRCC patients. A, B Multivariate Cox regression analysis of miRNA-187-3p and LRFN1 predicting PFS for patients with ccRCC from TCGA database. C, D Multivariate Cox regression analysis of miRNA-187-3p and LRFN1 predicting OS for patients with ccRCC from TCGA database. (TIF 214 KB) [file 12672_2022_523_MOESM2_ESM.tif]

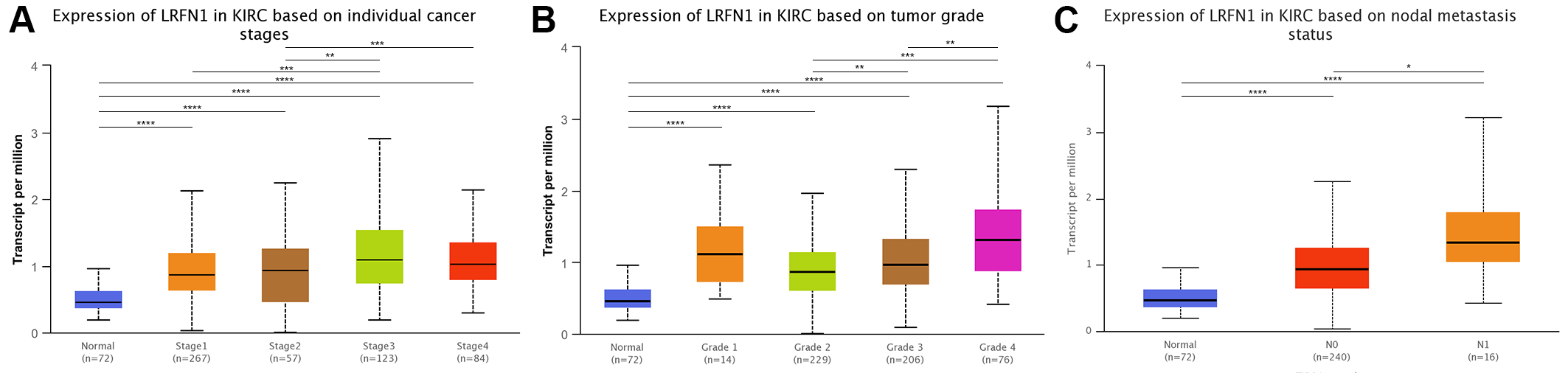

Supplement: Supplementary file 3 — Additional file 3 Figure S3. LRFN1 significantly correlated with advanced clinicopathological parameters for ccRCC patients. A, B Differential LRFN1 expression with tumor grade and individual clinical cancer stage for patients with ccRCC from TCGA database using unpaired t test. C Differential LRFN1 expression with nodal metastasis status for patients with ccRCC from TCGA database using unpaired t test. *P < 0.05; **P < 0.01; ***P < 0.001; ****P < 0.0001. (TIF 124 KB) [file 12672_2022_523_MOESM3_ESM.tif]
